# Intrathecal kappa free light chains as markers for multiple sclerosis

**DOI:** 10.1038/s41598-020-77029-7

**Published:** 2020-11-23

**Authors:** D. Vecchio, G. Bellomo, R. Serino, E. Virgilio, M. Lamonaca, U. Dianzani, R. Cantello, C. Comi, I. Crespi

**Affiliations:** 1 Clinical Biochemistry, Department of Translational Medicine, University of Piemonte Orientale, Novara, Italy; 2 Department of Translational Medicine, Neurology Unit, University of Piemonte Orientale, Corso Mazzini 18, 28100 Novara, Italy; 3 Interdisciplinary Research Center of Autoimmune Diseases (IRCAD), University of Piemonte Orientale, Novara, Italy; 4 Department of Health Sciences, University of Piemonte Orientale, Novara, Italy

**Keywords:** Neuroimmunology, Neurology

## Abstract

Cerebrospinal fluid (CSF) kappa free light chain (KFLC) index has been described as a reliable marker of intrathecal IgG synthesis to diagnose multiple sclerosis (MS). Our aims were: (1) to compare the efficiency of KFLC through different interpretation approaches in diagnosing MS. (2) to evaluate the prognostic value of KFLC in radiologically and clinically isolated syndromes (RIS-CIS). We enrolled 133 MS patients and 240 with other neurological diseases (93 inflammatory including 18 RIS-CIS, 147 non-inflammatory). Albumin, lambda free light chain (LFLC) and KFLC were measured in the CSF and serum by nephelometry. We included two groups of markers: (a) corrected for blood-CSF barrier permeability: immunoglobulin G (IgG), KFLC and LFLC indexes. (b) CSF ratios (not including albumin and serum-correction): CSF KFLC/LFLC, CSF KFLC/IgG, CSF LFLC/IgG. KFLC were significantly higher in MS patients compared to those with other diseases (both inflammatory or not). KFLC index and CSF KFLC/IgG ratio showed high sensitivity (93% and 86.5%) and moderate specificity (85% and 88%) in diagnosing MS. RIS-CIS patients who converted to MS showed greater KFLC index and CSF KFLC/IgG. Despite OB are confirmed to be the gold-standard to detect intrathecal IgG synthesis, the KFLC confirmed their accuracy in MS diagnosis. A “kappa-oriented” response characterizes MS and has a prognostic impact in the RIS-CIS population.

## Introduction

Kappa free light chains (KFLC) index has been described as a reliable marker of intrathecal immunoglobulin G (IgG) synthesis in multiple sclerosis (MS), resulting even more accurate than IgG index to discriminate MS from other neurological diseases^1–3^. Immunoglobulin free light chains (FLC), both of kappa and lambda subtypes, are produced by B-lymphocytes during antibody synthesis. An excess of FLC production in serum has been described in systemic inflammatory and autoimmune diseases^4^. Similarly, MS patients presented an overproduction limited to kappa chains in the cerebrospinal fluid (CSF)^5^. Since the CSF has a slower clearance of KFLC and IgG, in comparison to serum, we could measure these markers in the CSF to detect MS intrathecal synthesis of KFLC and IgG^6^. Primary aim of this work was to compare the efficiency of KFLC through different interpretation approaches in diagnosing MS. Other parameters included to discriminated MS were: oligoclonal bands (OB) detection, IgG (or Link) index and lambda FLC (LFLC) index. All these markers are corrected for blood-CSF barrier permeability: by comparison to serum for OB and by albumin ratio (serum/CSF albumin) for indexes^7^. Recently, the great sensitivity of intrathecal KFLC fraction towards MS diagnosis has been confirmed even using different approaches^8,9^. In this study we also considered measures for the excess of kappa and lambda FLC only in the CSF, called CSF ratios, that are not albumin and serum-corrected. These latter values were also investigated for a possible diagnostic role toward MS. The secondary aim of this work was to evaluate whether the above-mentioned markers had prognostic value in radiologically and clinically isolated syndromes (RIS-CIS) to identify which patients were at higher risk of conversion to MS.

## Methods

### Patients

We enrolled 406 consecutive patients who underwent a spinal tap during their diagnostic work-up for a neurological disorder between January 2015 and December 2019 at the Neurology Unit, University of Piemonte Orientale, Novara, Italy. They all gave written informed consent both diagnostic and research purposes (Ethical committee approval—Comitato Etico Interaziendale AOU "Maggiore della Carità" di Novara, ASL BI, ASL NO, ASL VCO: CE 190/19; all research was performed in accordance with relevant guidelines/regulations). We included in the present analysis 373 patients: 133 (88 females) MS according to McDonald criteria 2017^10^, 93 (50 females) with other neurological inflammatory diseases (ID) of the peripheral/central nervous system (CNS), and 147 (72 females) patients with non-ID neurological disorders. MS patients at diagnosis were classified as: 118 relapsing remitting, 12 secondary progressive and 3 primary progressive. ID included: RIS-CIS (N = 18 patients) according to McDonald criteria 2017^10^, isolated myelitis (N = 8 patients), acute demyelinating encephalomyelitis (N = 3), neuromyelitis optica spectrum disorders (N = 5), systemic autoimmune disorders with CNS involvement (N = 14), autoimmune encephalitis (N = 8), inflammatory neuropathies (N = 33 that were classified as acute/chronic inflammatory demyelinating polyneuropathies in 13 cases, antibody-mediated or in systemic autoimmune disorders in 20), acute cerebellitis (N = 3), Behcet syndrome (N = 1). Non-ID were: amyotrophic lateral sclerosis, dementia, non-inflammatory neuropathies, tumors. We excluded: 5 cerebral lymphomas, 16 CNS infectious diseases, 12 with no evidence of neurological disease at the end of the diagnostic work-up. Mean age of the included subjects was: 39.6 years (± standard deviation or SD 12.9) in MS, 51.1 (± 19.7) in ID and 57.2 (± 16.7) in non-ID.

### Laboratory

All sample were immediately processed. Albumin (N Antiserum to human albumin limit of quantitation (LoQ) was 0.17 mg/L; coefficient of variability (CV) *versus* low control = 4.3%, *versus* medium control = 3.6%, for CSF = 2.6%), IgG (N Antiserum to human immunoglobulin LoQ was 0.034 mg/L; CV *versus* low control = 3.4%, *versus* medium control = 2.1%, for CSF = 2.2%) and FLC (BNII Siemens Healthineers Diagnostic Products GmbH, Marburg, Germany; kit N latex FLC for kappa (LoQ was 0.0035 mg/dL) and lambda (LoQ was 0.01 mg/dL; CV *versus* control 1 = 1.9%, *versus* control 2 = 2.2%, for CSF = 3.4%) were measured by nephelometry evaluating absolute concentrations in CSF and serum, as previously described^2^. We calculated two groups of markers: (a) indexes (corrected for blood-CSF barrier permeability) that were IgG, KFLC and LFLC indexes. These values were calculated as follows using IgG index as example: CSF/serum IgG: CSF/serum albumin. The cut-off for KFLC index we employed was 5.0. This value showed the greatest combination of sensitivity and specificity in our population^2,3^. Regarding KFLC, other approaches have been studied to calculate the intrathecal fraction including different cut-offs of the index, Reiber's diagram, Presslauer's exponential curve, and Senel's linear curve^9^. As concerns MS diagnosis according to McDonald criteria 2017^10^ in our cohort, we then compared our cut-off for KFLC index^2^ to Reiber’s KFLC diagram^8^, since this latter approach presented the greater sensitivity in previous studies^9^. (b) CSF ratios (not albumin and serum-corrected): CSF KFLC/LFLC, CSF KFLC/IgG, CSF LFLC/IgG. Thirdly, OB were detected by isoelectrofocusing and immunoblotting (Hydragel 1–3 o Hydragel 1–9 CSF Isofocusing on Hydrasys, Sebia, Bagno a Ripoli, Firenze, Italia) according to standard methods^10^. The gel was evaluated by two independent operators for the presence of OB and for the attribution of one of the five patterns according to Freedman^11^. Type II (presence of OB exclusively in CSF) and III (presence of OB in both CSF and serum but clear prevalence of CSF) were considered positive for intrathecal IgG synthesis.

### Statistical analysis

Continuous variables were expressed with mean and SD. Their distributions were checked with Shapiro–Wilk test and resulted not normally distributed. To compare data of multiple groups (MS, ID and NID patients), a non-parametric ANOVA (Kruskal–Wallis analysis) was applied with Bonferroni correction for multiple comparisons (p-values below 0.005 were considered to be significant). Sensitivity was calculated as “true-positive/(true-positive + false-negative)”, specificity as “true-negative/(true-negative + false-positive)”. Area under curve (AUC), sensitivity and specificity were performed on received-operating curve (ROC) using a VassarStat software and with a Bayesian calculator made available by The Italian Society of Laboratory Medicine (SIPMEL). Differences between patients with RIS-CIS, that converted to MS, and those who did not covert were explored by Mann–Whitney test. The prognostic value of KFLC was determined by comparing converters *versus* non-converters by binary logistic regression analyses. P-values below 0.05 were considered to be significant.

### Ethics approval

Local Ethical committee approval (Comitato Etico Interaziendale AOU "Maggiore della Carità" di Novara, ASL BI, ASL NO, ASL VCO): CE 190/19.

### Consent to participate/consent for publication

Written consent obtained from all participants.

## Results

Data are shown in Table 1 (N. of included patients: 373).

**Table 1:** Absolute concentrations of kappa (K) and lambda (L) free light chains (FLC), CSF ratios and indexes were determined in multiple sclerosis (MS), inflammatory neurological diseases other than MS (ID), and non-ID.

	MS (n = 133)	ID (n = 93)	Non-ID (n = 147)	Sensitivity (%)	Specificity (%)
**CSF markers (not albumin or serum-corrected): mean ± SD**					
KFLC (mg/dl)	0.48 ± 0.56*#	0.20 ± 0.53	0.03 ± 0.13	78.9	97.5
LFLC (mg/dl)	0.13 ± 0.16	0.05 ± 056	0.06 ± 0.22	56.3	81.8
IgG (mg/dl)	4.90 ± 3.24	6.64 ± 12.71	3.91 ± 6.28	60.9	62.1
KFLC/LFLC ratio	16.24 ± 40.47	8.31 ± 33.37	0.98 ± 0.53	77.8	77.5
KFLC/IgG ratio	85.44 ± 66.67*#	33.36 ± 57.08	9.20 ± 7.49	86.5	87.9
LFLC/IgG ratio	2.81 ± 2.86*	1.17 ± 0.99	1.17 ± 0.83	51.3	88.1
**Markers corrected for blood-CSF barrier permeability**					
IgG index: mean ± SD	0.85 ± 0.46*	0.56 ± 0.22	0.50 ± 0.13	70.5	68.8
KFLC index: mean ± SD	70.84 ± 86.70*#	26.38 ± 77.25	3.08 ± 6.21	93.2	85.4
LFLC index: mean ± SD	17.07 ± 23.00*	3.93 ± 6.88	3.76 ± 8.43	80.3	80.3
Reiber’s KFLC diagram: mean ± SD	88.2 ± 18.2*#	34.5 ± 22.10	16.4 ± 22.6	98.1	53.2
OB: yes/no	127/6	28/65	7/140	95.5	85.2

Values are expressed in mean ± standard deviation (SD). We included 373 patients for KFLC and oligoclonal bands (OB) evaluation, 223 of them were tested also for LFLC.

OB yes/no: “yes” was intended for types II (OB exclusively in CSF and not in serum) and III (contemporary presence of OB in both CSF and serum with a clear predominance in CSF).

*Means significantly different in MS from ID and non-ID (p < 0.005 was considered significant according to Bonferroni correction for multiple comparisons).

^#^Means significantly different among three groups (MS *versus* ID *versus* non-ID, p < 0.05).

KFLC differentiated MS patients from those with ID and non-ID (p < 0.005). In fact, KFLC index and CSF KFLC/IgG ratio were significantly higher in MS then in other neurological conditions. Similarly, MS patients presented increased absolute concentrations of KFLC (mean value was 0.48 mg/dl) compared to both ID (0.20 mg/dl) and non-ID patients (0.03). The KFLC, despite considering different interpretation approaches, permitted also to distinguish among the three groups (MS *versus* ID *versus* non-ID). Conversely, LFLC were not relevant in MS diagnosis (223 patients of the 373 included were tested for LFLC). LFLC index and CSF LFLC/IgG ratio resulted greater in MS then in other neurological conditions, but did not differ significantly among the three groups.

KFLC index emerged as the most sensitive marker corrected for blood-CSF barrier permeability in diagnosing MS. Its sensitivity of 93% overtook that of IgG index (70.5%), and was only slightly lower than of OB (95.5%). Accordingly, we confirmed the greater accuracy of OB in MS diagnosis, according to McDonald criteria 2017^10^. Of note, in our study the specificity of OB was similar to that of KFLC index (85%). If comparing different approaches to calculate KFLC intrathecal fraction in our cohort, sensitivity towards MS diagnosis was 98% for Reiber’s KFLC diagram^9^, in face of 53% specificity. Thus, concerning MS diagnosis, KFCL index performances resulted more similar to that of OB in our population. Among CSF markers, only KFLC/IgG ratio resulted a sensitive marker of intrathecal IgG synthesis (sensitivity 86.5%).

**Figure 1:**
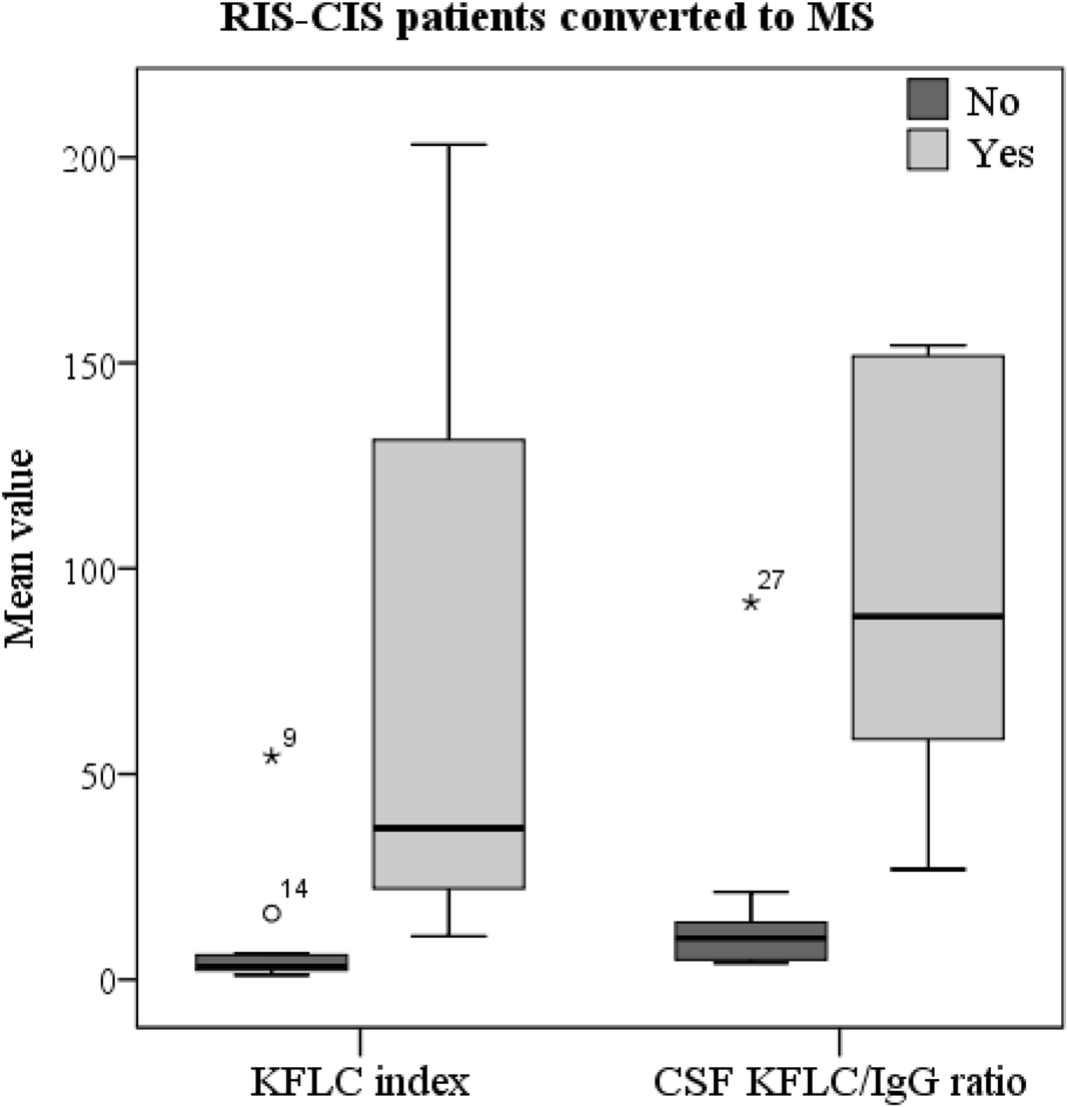
Kappa free light chain (KFLC), expressed through different interpretation approaches, in patients with radiological or clinically isolated syndrome (RIS-CIS) according to conversion to multiple sclerosis (MS) after a minimum follow up of one year. Mean values were: CSF KFLC/IgG ratio 71.5 (± SD 45.9) in RIS-CIS converted to MS *versus* 26.0 (± 28.8) in non-converted (p = 0.003); KFLC index 38.2 (± 63.5) *versus* 5.9 (± 10.8) (p = 0.03). Mean values were: CSF KFLC/IgG ratio 71.5 (± SD 45.9) in RIS-CIS converted to MS *versus* 26.0 (± 28.8) in non-converted (p = 0.003); KFLC index 38.2 (± 63.5) versus 5.9 (± 10.8) (p = 0.03). No difference in LFLC was found between RIS-CIS patients with and without conversion to MS. CSF KFLC/IgG ratio resulted more informative in detecting whose patients were at risk of convert to MS. In fact, RIS-CIS patients with elevated CSF KFLC/IgG ratio had a higher risk to convert to MS (hazard ratio, HR 1.05; 95% CI 1.01–1,10; p = 0.02). Conversely, regression was not significant for KFLC index (HR 1.07; 95% CI 0.99–1.16; p = 0.09).

In our study, we included 3 patients with RIS and 15 with CIS. Mean age of the 18 subjects (11 females) was: 36.3 years (± SD 8.5). CIS presentations included: unilateral optic neuritis (6 patients), focal supratentorial syndrome (4), and partial myelopathy (5). Brain and spinal magnetic resonance (performed at the time of diagnostic work-up) did not fulfilled criteria for dissemination in space in 12 cases, and for dissemination in time in the remaining 6. Six (33%) patients converted to MS during the follow up (that lasted at least one year), developing new lesions over time. Mean follow up of this subgroup of 18 subjects was 3.6 years (± SD 3.6). All the subjects that converted to MS presented OB and significantly higher KFLC then those who remained RIS-CIS (Fig. 1).

Gender and age at onset did not differ significantly among RIS-CIS patients who converted or not to MS. Those patients who presented with optic neuritis converted less to MS then other types of onset (RIS, focal supratentorial syndrome, or partial myelopathy) (p = 0.07).

## Discussion

Our study confirmed the role of KFLC in the diagnostic work-up for MS. Both KFLC index (corrected for blood-CSF barrier permeability) and KFLC/IgG ratio (evaluating the overproduction of KFLC in CSF only) showed a high sensitivity and decent specificity towards MS diagnosis. Overall, OB remained the gold standard for CSF analysis in MS.

These results still supported our routine testing for CSF analysis using a KFLC index to collect all cases suspected for MS, and proceeding with OB detection only if KFLC index is higher than 5. If compared to by isoelectrofocusing and immunoblotting, KFLC index measurement has some advantages: it can be completely automatized, it is operator-independent in interpretation, less time-consuming and less expensive^2^. Recently, the great sensitivity of intrathecal KFLC fraction has been confirmed even using several approaches such as: different ROC-curve determined KFLC index cut-offs, Reiber's diagram, Presslauer's exponential curve, and Senel's linear curve^8,9,12^. Schwenkenbecher et al. showed that Reiber’s diagram had a greater sensitivity towards intrathecal Ig synthesis (if compared with the above-mentioned approaches and to a KFLC index cut-off of 5.9)^9^. In our cohort we confirmed the greatest sensitivity (98%) of Reiber’s KFLC diagram toward MS. Although, this measure lacked of specificity in our population. Senel et al. calculated the CSF-serum ratio of KFLC, named Q KFLC (applying CSF-serum albumin ratio-dependent reference values). They showed no relevant difference for MS diagnostic accuracy comparing Q KFLC to the ROC curve determined cut-off value in 1224 patients^12^. We employed KFLC index according to its sensitivity of 93% and specificity of 85% in our population. Moreover, “false positive” values for KFLC index were double-check for the clinical diagnosis at the end of diagnostic work-up and after a follow up of one year. In our study, we considered another approach to evaluated the excess of intrathecal KFLC only in CSF, and not corrected for blood-CSF barrier permeability. This CSF KFLC/IgG ratio also resulted sensitive (and specific) in discriminating MS from other neurological conditions, as previously described^13^. Not only this marker could be used to search for intrathecal IgG synthesis in suspect MS if serum is not available, but also supported the hypothesis that MS patients have an “excess” of KFLC production limited to the CSF^14^. These data confirmed the “kappa-oriented” immune reaction in MS CSF^14,15^. To our knowledge, the KFLC overproduction in MS patients has not been clarified yet. Increased concentrations of serum FLC have been described in several autoimmune disorders (and related to disease activity in few) in relation to the phenomenon of “antigen excess”^4^. Although not explaining the “kappa” prevalence, this mechanism could be speculated for CSF in the MS population, and have prognostic relevance.

The presence of OB during the early MS phases have been discussed also as a negative prognostic indicator for disease outcome^16,17^ and we previously reported KFLC index being a significant predictor for disability over time being higher in those patients who developed greater disability in the short term^18^. Robust data have been published on the role of OB in predicting CIS conversion to MS^19^. In this study we included a small group of RIS-CIS patients and evaluated conversion to MS in the short term. KFLC through different interpretation approaches resulted higher in those subjects who converted to MS during the follow up, being CSF KFLC/IgG ratio more significant then KFLC index. A prognostic value for KFLC have been discussed in few recent studies^20,21^. Villart et al. associated high CSF KFLC absolute concentrations (categorized *versus* less than 0.53 mg/l) to a greater probability of conversion to MS in 78 CIS patients^22^. A similar prognostic role was confirmed for KFLC index by Makshakov et al.^23^. There is no prognostic data on the excess of KFLC in the CSF (using the ratio that includes CSF IgG as we did). Moreover, in our study, the CSF KFLC/IgG ratio better stratified the risk of conversion to MS if compared to KFLC index. LFLC did not differ among the groups, as previously described^21^. Early conversion to MS was less frequent with optic neuritis onset, whereas other clinical/paraclinical parameters failed to identify converters in our cohort (possibly because of the small sample size). Senel et at. enrolled 77 CIS patients according to Mc Donald 2010 criteria^24^, of whom 38 converted to MS. They showed that KFLC are predictors for conversion to MS (almost as sensitive as OB)^25^. In the present study, the application of McDonald criteria 2017 reduced the number of cases that could be classify as RIS-CIS, and definitely, a prolonged follow up with long-term outcomes could improve the prognostic role of KFLC.

Another limit of this study was that our patients underwent a unique lumbar puncture. Consequently, we do not have data on any changes of FLC levels over time, despite foregoing reports suggest they remain stable^26^. It is questionable whether KFLC could change with treatment particularly targeting B-cells.

In conclusion, we confirmed KFLC index as the most sensible and specific quantitative marker for diagnosing MS and suggested that CSF KFLC/IgG might be employed to find whose RIS-CIS patients will convert to MS.

## Data Availability

Data available on request.
